# Rapid Determination of Sotolon in Fortified Wines Using a Miniaturized Liquid-Liquid Extraction Followed by LC-MS/MS Analysis

**DOI:** 10.1155/2018/4393040

**Published:** 2018-12-16

**Authors:** Vanda Pereira, João M. Leça, João M. Gaspar, Ana C. Pereira, José C. Marques

**Affiliations:** ^1^Faculty of Exact Sciences and Engineering, University of Madeira, Campus da Penteada, 9020-105 Funchal, Portugal; ^2^Institute of Nanostructures Nanomodelling and Nanofabrication (I3N), University of Aveiro, 3810-193 Aveiro, Portugal; ^3^CIEPQPF, Department of Chemical Engineering, University of Coimbra, Pólo II-Rua Sílvio Lima, 3030-790 Coimbra, Portugal

## Abstract

Sotolon (4,5-dimethyl-3-hydroxy-2,5-dihydrofuran-2-one) is a powerful odorant usually pointed out as being responsible not only for the characteristic curry notes of the finest fortified wines but also for the off-flavour notes in prematurely oxidized white wines. Most methods reported in literature for quantifying sotolon in wines are quite laborious and use large volumes of organic solvents. Thus, in the present study, the development of a simple, fast, and environment-friendly method for the quantification of sotolon in fortified wine is herein presented. The proposed method uses a single-step liquid-liquid extraction followed by RP-LC-MS/MS and was optimized using a full factorial design. The method showed good linearity (*R*^2^ = 0.9999), intra- and interday precision lower than 10% RSD, recovery of about 95%, and high sensitivity (LOQ of 0.04 *μ*g/L). The method was applied to analyse 44 fortified wines from different styles (from dry to sweet wines) and ages (3–115 years old), and it was found that it covers the concentration range usually found for this compound in this kind of alcoholic beverages, which was found to be within 6.3–810 *μ*g/L. Thus, it can be concluded that this method can be used as an accurate tool for the rapid analysis of sotolon, since the early stages of its formation up to long ageing periods.

## 1. Introduction

Sotolon (PubChem CID: 62835) is a well-known odorant, with a powerful sweet/honey/nutty/walnut/spicy/curry/burnt scent, depending on its concentration and enantiomeric distribution [1–4]. Sotolon is responsible for the distinctive aroma of several foodstuffs, imparting a curry odour to fenugreek seeds [5], a sweet aroma to raw cane sugar [6], burnt notes to aged sake [7], a seasoning-like aroma to roasted coffee [8] and soy sauce [9], a curry/liquorice scent to sherry vinegar [10], and a caramel-like aroma to sweet-type Chinese rice wine [11]. Additionally, it has been found that sotolon plays an important role in the aroma of botrytized wines such as Tokay and Sauternes [12–14], vin jaune (“yellow wine”) from Jura [3, 15], Scheurebe and Gewürztraminer white wines [16], Chinese Syrah wine [17], and fortified wines such as vin doux naturels (VDN) [18, 19], port [4], Madeira [20], sherry [21–23], and Polish Jutrzenka [24]. In fortified wines, sotolon is usually quantified well above its odour threshold, namely, 8 *µ*g/L in dry white wine and 19 *μ*g/L in port wine [1, 4], at concentrations up to 2000 *µ*g/L in Madeira wines [20]. On the contrary, sotolon's off-flavour character, associated to the premature oxidative ageing (premox), has been found to overlap the expected fruity, flowery, and freshness of white wines [1, 25, 26], being found at a concentration usually lower than 140 *μ*g/L [27]. Several precursors and pathways have been proposed for the occurrence of sotolon in wines: by peroxidation of acetaldehyde [28], thermally produced from intermediates generated from the Maillard reaction such as pyruvic and ketoglutaric (via glutamic acid) acids [29], by enzymatic or chemical deamination of threonine followed by the aldol condensation between *α*-ketobutyric acid and acetaldehyde [30, 31], and by oxidative degradation of ascorbic acid in the presence of ethanol [32]. Other Maillard-type reactions are also known to produce sotolon in thermally processed model systems composed by glucose/cysteine, rhamnose/cysteine [33], hydroxyacetaldehyde/diacetyl [4, 32], and pyruvic acid/glycine [34]. Despite the main formation pathways not being consensual, it is generally accepted that wine oxidation, storage time, temperature, and sugar concentration are associated with sotolon development. In fact, simple sugars may play a central role on its formation. Recently, Pereira, Santos, Cacho, and Marques [35] identified sotolon in thermally processed fructose model wines (125 g/L of fructose dissolved in a 18% ethanolic solution with 6 g/L of tartaric acid and pH 3.5) and suggested that fructose thermal degradation, in the acidic medium, by itself, may be responsible for much of the sotolon production in sweet fortified wines.

The main difficulty to quantify sotolon in wines is related to its low concentration, and therefore, a preconcentration step is needed. Also, sotolon has a high boiling temperature (184°C), which makes it difficult to analyse by headspace sampling (dynamic headspace (DHS) and solid-phase microextraction (SPME)). The analysis methods reported in literature for quantifying sotolon in wines are quite laborious, usually using classical liquid-liquid extraction (LLE) or solid-phase extraction (SPE) followed by gas (GC) or liquid (LC) chromatographic separation and mass spectrometry (MS or MS/MS) and UV/Vis spectroscopy (DAD) detection [26, 36–39]. Other authors proposed the use of microextraction by packed sorbent followed by ultrahigh-pressure liquid chromatography (MEPS/UHPLC-PDA) [40], which without automatization is quite laborious. In fact, current trends and challenges in sample preparation not only look toward the smaller sample and reagent consumption but also toward the simplification and shortening of the experimental procedures.

In this study, a new method is proposed for the simple and rapid quantification of sotolon in fortified wines. A single-step extraction method was optimized using an experimental design and its performance was evaluated. This new method was tested to quantify sotolon in several fortified wines.

## 2. Materials and Methods

### 2.1. Chemicals and Samples

All chemicals and standards had a purity grade higher than 97%. Sotolon was purchased from SAFC (St. Louis, MO, USA). Absolute ethanol was from Sigma-Aldrich (Steinheim, Germany); formic acid, tartaric acid, and UPLC grade methanol were from Panreac (Barcelona, Spain). Ethyl acetate was from Fisher Scientific (Leicestershire, UK). The type 1 ultrapure water was obtained with a Simplicity® UV apparatus from Millipore (Milford, MA, USA).

A synthetic fortified wine was prepared and consisted in a solution containing 6 g/L of tartaric acid in a 180 mL/L ethanol/water solution, pH adjusted to 3.5 with a 1 M sodium hydroxide (Panreac) solution. Standard stock (400 mg/L) and working (200 mg/L) solutions of sotolon were rigorously prepared in ethanol and water, respectively. Seven calibration points were prepared in synthetic wine and in a fortified wine, within the validation range 1–2000 *μ*g/L.

All eluents were filtered through a hydrophilic polypropylene 0.2 *μ*m pore size membrane filter (Pall Corporation, Ann Arbor), before being used. Wine extracts were filtered using Chromafil PTFE 0.2 *µ*m syringe filters (Macherey-Nagel, Düren, Germany), before being analysed.

A sample set of 44 fortified wines with different ages (3–115 years old), sweetness degrees (including dry (5), medium-dry (6), medium-sweet (10), and sweet wines (23)), and ethanol contents (18 to 20%) were analysed.

### 2.2. Extraction Optimization

The biggest challenge was how to discriminate and determine the trace quantities of sotolon from a complex matrix without using large volumes of toxic organic solvents and time-consuming procedures. A QuEChERS (quick, easy, cheap, effective, rugged, and safe) experimental procedure was firstly tested to extract sotolon from fortified wines since it has been widely applied to various classes of compounds in several matrices [41]. Thus, the following procedure was firstly tested: 10 mL of wine, placed into 50-mL PTFE centrifuge tubes, 4 mL of acetonitrile, 1 g of sodium citrate tribasic dehydrate, 500 mg sodium citrate dibasic sesquihydrate, 1 g of sodium chloride, and 4 g of anhydrous magnesium sulphate were added. The tubes were vortexed for 5 min and centrifuged for 5 min at 4400 rpm (Centrifuge Eppendorf 5702, NY, USA). The organic layer was evaporated under a moderate nitrogen stream. Ethyl acetate was also tested in the same conditions. Then, the salts and buffers were removed from the QuEChERS extraction procedure because it was verified that their addiction decreases the extraction yield of sotolon, simplifying the procedure. Consequently, the developed protocol was then nominated as miniaturized liquid-liquid extraction and further validated.

Full factorial design was then used to perform the optimization of the extraction procedure. Two experimental variables at 3 levels were chosen: the sample volume (8, 10, and 15 mL) and the extractant volume (4, 5, and 8 mL). All sample/extractant volume combinations were randomly tested. For this study, a commercial fortified wine was used. The data analysis was performed using the Matlab software, version R2016b, to estimate the best combination of the sample and extractant volume to maximize the response of sotolon in LC-MS/MS analysis.

### 2.3. Apparatus and Chromatographic Conditions

The LC-MS/MS system was from Shimadzu (Kyoto, Japan) and is composed by a Nexera X2 UHPLC system with a binary LC-30AD pumps, a DGU-20 A5 degassing unit, a CTO-20A column oven, a SIL-30AC autosampler, and a triple-quadruple mass spectrometer LCMS-8040, equipped with an ESI ionization module. Purified nitrogen (Genius 1050 nitrogen generator, Peak Scientific, Inchinnan, Scotland, UK) was used as a drying gas.

Sample extracts were firstly separated in a reversed-phase (RP) column using a Kinetex C18 column, 150 × 2.1 mm, 2.6 *μ*m, 100 Å, from Phenomenex (Torrance, CA, USA) thermostated at 40°C. The injection volume was 5 *μ*L. Each sample extract was injected twice, while standard extracts were injected three times. The gradient elution was optimized with an extract of a fortified wine sample to obtain the best separation and peak resolution, ensuring good selectivity and sensitivity at the retention time 5.1 min. The chromatographic separation was then performed using a linear gradient elution, as follows: 5% A for 4 min and then increased up to 30% A in 2 min, then up to 100% A in 1 min, being reduced to 5% A in 3 min and held at 5% A for 5 min, with the flow rate set to 0.4 mL/min, methanol as solution A, and acidified water (0.1% formic acid) as solution B. The total run time was 15 min per sample, but the column eluent was only directed towards the detector between the 0.5 and 9.0 min. MS detection was carried out using ESI in the positive ionization mode, and the optimized conditions were as follows: the desolvation line temperature was maintained at 250°C and the block heater at 400°C, while the nebulizing gas flow was set to 2.5 L/min and the drying gas flow to 17.5 L/min. Sotolon was analysed in the multiple reaction-monitoring (MRM) mode, 145 using the following transition: 129.1 *m/z* ⟶ 55.1 *m/z* (quantification) and 129.1 *m/z* ⟶ 83.0 *m/z* (identification). The optimal collision energy (−18 eV) was optimized by the direct injection of a standard solution of sotolon (10 mg/L) into the LC-MS/MS system and by performing various tests, automatically programmed by the Labsolutions 5.7 software. The data acquisition and peak integration processing were both performed with Labsolutions 5.7 software from Shimadzu.

## 3. Results and Discussion

LC-MS/MS combined with MRM was selected for the analysis of sotolon in fortified wines due to its high loading capacity, sensitivity, and selectivity [42], which is quite important when analysing compounds that usually occur at very low quantities (ppb levels) in complex matrices such as fortified wines. LC-MS has already been used to quantify sotolon in wines [36], but using a laborious and time-consuming extraction procedure, also using a large amount of the organic solvent. Thus, this study intends to simplify the extraction procedure, miniaturizing the use of organic solvents.

### 3.1. Extraction Optimization

As aforementioned, acetonitrile and ethyl acetate extraction performances were investigated. Ethyl acetate was then chosen as the best extractant solvent for sotolon in fortified wines considering that the chromatograms revealed no interferences and less background noise. Also, the addition of salts and buffers was ascertained, and the results obtained indicated that their addition decreased the extraction yield of sotolon, and therefore, both were removed from the extraction procedure, becoming an extraction procedure even cheaper and easier to use. According to previous studies [43], a full factorial design was then carried out to optimize the extraction procedure, considering three levels per factor. Two factors were considered: the sample (8, 10, and 15 mL) and extractant (4, 5, and 8 mL) volumes. These two variables revealed to be significant, as well as the interaction factor between them (*p* values lower than 0.05).

**Figure 1:**
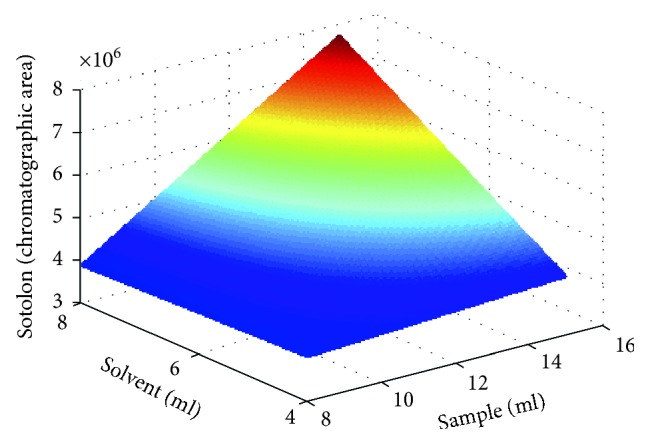
Results obtained using the design of experiment to optimize the single-step extraction procedure, with sample and solvent extraction volumes as variables. The colormap illustrates the variation of the LC-MS/MS response of sotolon.

Figure 1 illustrates that sotolon's chromatographic peak areas can be maximized when higher volumes of sample and organic solvent are used to perform the extraction procedure.

### 3.2. Final Extraction Procedure

Fifteen microliters of the sample and 8 mL of ethyl acetate (extractant solvent) were placed into 50 mL PTFE centrifuge tubes. The tubes were then vortexed for 5 min and centrifuged at 4400 rpm for 10 min. The upper phase was collected and evaporated using a small nitrogen flow. The residue was redissolved with 0.1% formic acid up to a final volume of 1 mL and filtered through Chromafil PTFE 0.2 *µ*m syringe filters (Macherey-Nagel, Düren, Germany). Each sample/standard solution was extracted twice. Finally, 5 *µ*L of sample extract was injected into the LC-MS/MS system.

### 3.3. Performance Evaluation of the Method

To check the method performance, the following parameters were evaluated: selectivity, matrix effect, linearity, sensitivity, precision, and accuracy.

**Figure 2:**
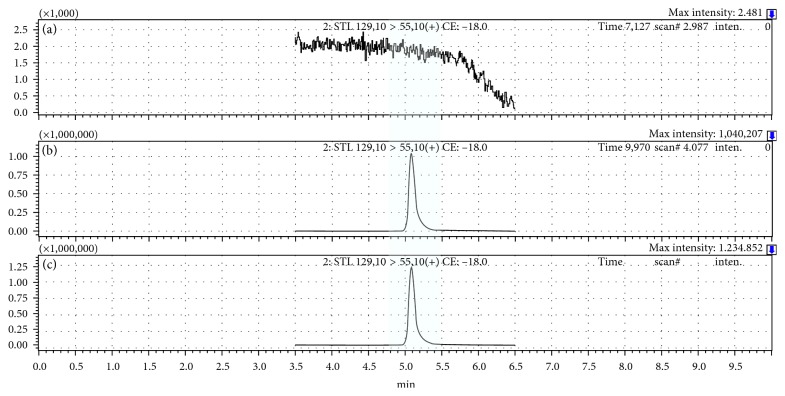
Typical chromatograms of synthetic wine (a), a standard calibration solution of 250 *µ*g/L (b), and a fortified wine (c) using the proposed single-step extraction procedure.

The methodology showed good selectivity, since it verified the absence of interferences at the sotolon retention time of synthetic and real wine samples, as depicted in Figure 2. Initially, five fortified wines were analysed to ensure selectivity, and no chromatographic interferences were verified at the retention time of sotolon. After the method performance evaluation was conducted, the selectivity was later confirmed through the analysis of 44 fortified wines of different ages and sweetness degrees.

Then, the matrix effect (ME) was evaluated by the slope comparison method [44, 45], obtaining the percentage of the quotient between the slopes of the curve obtained by spiking synthetic wine with different amounts of pure sotolon standard addition and the curve obtained by spiking a fortified wine with the same concentrations, using the following equation:

(1) $$\begin{array}{c} \text{\% ME }=\left[\frac{\left(\text{slope of the fortified wine curve }-\text{ slope of the synthetic wine curve }\right)}{\text{slope of the synthetic wine curve }}\right]\times 100. \end{array}$$

Curves were obtained by plotting the sotolon peak area against the corresponding concentration, between 25 and 200 *μ*g/L, and no obvious matrix effect was encountered (ME = 13%). Then, standard solutions were prepared at 7 concentration levels of sotolon in synthetic wine (3 extracts for each level): 1, 10, 25, 50, 125, 1000, and 2000 *μ*g/L, and external standard calibration method was adopted. The calibration curve was plotted, and the correlation coefficient (*R*^2^) was determined to check the method linearity. A good correlation coefficient was obtained, *R*^2^ = 0.9999, confirming the linearity of the method. Sensitivity was also evaluated, determining the limit of detection (LOD) and the limit of quantification (LOQ) with signal-to-noise (S/N) ratio greater than 3 and 10, respectively. The method revealed great sensitivity, since that LOD and LOQ results (Table 1) are quite below the odour threshold of sotolon found in wines and comparable or even lower to most recent methods found in literature [26, 36, 37, 40, 46].

Repeatability (intraday) and reproducibility (interday) of two standard solutions and one fortified wine were used to evaluate the method precision. Intraday RSD (%) was assessed by the response of 10 successive analyses, while interday RSD (%) was checked by the results of 5 analyses of the same samples in 3 different days. Good results were obtained because repeatability and reproducibility never exceeded 10% of RSD.

The method accuracy was evaluated through recovery experiments, spiking a fortified wine, with known amounts of sotolon at two representative concentrations levels (250 and 1000 *μ*g/L), within the calibration range.

Recovery calculations were obtained by comparison of the mean values of the 3 replicates with theoretical concentrations of each one. The proposed method revealed to be accurate, considering that recovery results were higher than 92%, demonstrating the accuracy of the method.

### 3.4. Analysis of Real Samples

Regarding the applicability of the proposed method for the sotolon determination in fortified wines, a set of real samples, comprised by 44 fortified wines, was analysed. Good resolution and selectivity were obtained, as depicted on Figure 2.  Table 2 shows that the quantification results varied between 6.3 ± 0.4 and 810 ± 20 *μ*g/L. These results confirm the applicability of the proposed method to quantify sotolon in fortified wines, covering the concentration range usually found in this matrix. A correlation was found between the sotolon values and the age of sweet wines (*R*^2^ = 0.8887), while in the other styles, no correlation was found. On the contrary, it is interesting to notice that the sweeter wines (sweet and medium-sweet), which are more than 10-years-old, hold in average 505 *μ*g/L, while the dryer ones present almost half (282 *μ*g/L). Moreover, it was found that most fortified wines (89%) presented concentration levels quite above the odour threshold of sotolon in port wine (19 *μ*g/L), namely, up to 43-fold above, in the oldest wine.

## 4. Conclusions

The single-step liquid-liquid extraction followed by LC-MS/MS analysis herein proposed reveal to be a simple, efficient, reliable, and sensitive method for the determination of sotolon in fortified wines, in less than 15 min for sample preparation and 15 min analysis run time. The experimental design employed enabled the optimization of the extraction, ensuring a good compromise between the LC-MS response and the reduction of the volumes of the sample and extractant solvent. It was shown that the method calibration can be done using the synthetic wine as an effective matrix. The methodology shows good linearity, sensitivity, selectivity, precision, and accuracy, minimizing the use of high volumes of sample and organic solvents. The applicability of the method was demonstrated through the analysis of a set of 44 fortified wines and covered the concentration range usually found for this compound in this alcoholic beverage. Most wines presented concentration levels quite above the odour threshold, and a correlation was found between the sotolon values and the age of sweet fortified wines.

## Figures and Tables

**Table 1:** Performance results obtained for the proposed miniaturized liquid-liquid extraction method.

Parameter	Result
Linear regression	*A*_STL_ = 46437 [STL] + 43722
Linear concentration range (*μ*g/L)	1.0–2000
*R* ^2^	0.9999
LOD (*μ*g/L)	0.011
LOQ (*μ*g/L)	0.037

Repeatability (% RSD)	3.4–6.4
Reproducibility (% RSD)	5.4–10.0

Recovery (%)	
FW + STL 250 *μ*g/L	92
FW + STL 1000 *μ*g/L	98

A_STL_, sotolon peak area; [STL], sotolon concentration in *μ*g/L; LOD, limit of detection; LOQ, limit of quantification; FW, fortified wine; STL, sotolon.

**Table 2:** Application of the proposed method for the quantification of sotolon (expressed in the mean value ± standard deviation) in 44 fortified wines of different ages and sweetness degrees.

FW	Style	Age	Sotolon (*µ*g/L)	FW	Style	Age	Sotolon (*µ*g/L)
1	Sweet	—	8.3 ± 0.9	23	Sweet	21	697 ± 20
2	Sweet	3	6.3 ± 0.4	24	Medium-sweet	—	550 ± 13
3	Sweet	3	9.6 ± 0.4	25	Medium-sweet	—	398 ± 28
4	Sweet	3	83 ± 1	26	Medium-sweet	11	137 ± 5
5	Sweet	3	66.2 ± 0.6	27	Medium-sweet	20	283 ± 30
6	Sweet	3	62 ± 3	28	Medium-sweet	22	346 ± 20
7	Sweet	3	65 ± 3	29	Medium-sweet	51	623 ± 27
8	Sweet	3	63.1 ± 0.5	30	Medium-sweet	55	487 ± 75
9	Sweet	3	17 ± 1	31	Medium-sweet	87	739 ± 49
10	Sweet	3	18.0 ± 0.9	32	Medium-sweet	97	393 ± 25
11	Sweet	5	145 ± 6	33	Medium-sweet	115	810 ± 20
12	Sweet	5	173 ± 6	34	Medium-dry	—	494 ± 87
13	Sweet	5	254 ± 7	35	Medium-dry	—	274 ± 10
14	Sweet	5	320 ± 4	36	Medium-dry	5	176 ± 19
15	Sweet	5	111 ± 3	37	Medium-dry	20	283 ± 13
16	Sweet	6	264 ± 16	38	Medium-dry	22	142.0 ± 0.4
17	Sweet	7	214 ± 2	39	Medium-dry	40	417 ± 9
18	Sweet	9	268 ± 13	40	dry	-	427 ± 15
19	Sweet	13	493 ± 5	41	dry	-	186 ± 1
20	Sweet	17	509 ± 24	42	dry	12	113 ± 8
21	Sweet	18	645 ± 8	43	dry	27	242 ± 12
22	Sweet	19	461 ± 15	44	dry	38	346 ± 3

## Data Availability

The data used to support the findings of this study are included within the article.
